# Regression analysis on forward modeling of diffuse optical tomography system for carcinoma cell detection

**DOI:** 10.1038/s41598-023-29063-4

**Published:** 2023-02-10

**Authors:** K. Uma Maheswari, M. Thilak, N. SenthilKumar, N. Nagaprasad, Leta Tesfaye Jule, Venkatesh Seenivasan, Krishnaraj Ramaswamy

**Affiliations:** 1 Department of Electronics and Communication Engineering, SRM TRP Engineering College, Trichy, India; 2 Department of Mechanical Engineering, SRM TRP Engineering College, Trichy, India; 3 Department of Mechanical Engineering, ULTRA College of Engineering and Technology, Madurai, 625 104 Tamil Nadu India; 4 Department of Physics, College of Natural and Computational Science, Dambi Dollo University, Dembi Dolo, Ethiopia; 5 Centre for Excellence-Indigenous Knowledge, Innovative Technology Transfer and Entrepreneurship, Dambi Dollo University, Dembi Dolo, Ethiopia; 6 Department of Mechanical Engineering, Sri Eshwar College of Engineering, Coimbatore, India; 7 Department of Mechanical Engineering, College of Engineering and Technology, Dambi Dollo University, Dembi Dolo, Ethiopia

**Keywords:** Cancer, Cell biology, Computational biology and bioinformatics, Drug discovery, Diseases, Health care, Medical research

## Abstract

The forward model design was employed in the Diffuse Optical Tomography (DOT) system to determine the optimal photonic flux in soft tissues like the brain and breast. Absorption coefficient (mua), reduced scattering coefficient (mus), and photonic flux (phi) were the parameters subjected to optimization. The Box–Behnken Design (BBD) method of the Response Surface Methodology (RSM) was applied to enhance the Diffuse Optical Tomography experimental system. The DC modulation voltages applied to different laser diodes of 850 nm and 780 nm wavelengths and spacing between the source and detector are the two factors operating on three optimization parameters that predicted the result through two-dimensional tissue image contours. The analysis of the Variance (ANOVA) model developed was substantial (R^2^ =  > 0.954). The experimental results indicate that spacing and wavelength were more influential factors for rebuilding image contour. The position of the tumor in soft tissues is inspired by parameters like absorption coefficient and scattering coefficient, which depend on DC voltages applied to the Laser diode. This regression method predicted the values throughout the studied parameter space and was suitable for enhancement learning of diffuse optical tomography systems. The range of residual error percentage evaluated between experimental and predicted values for mua, mus, and phi was 0.301%, 0.287%, and 0.1%, respectively.

## Introduction

The brain and soft breast tissues of the human body are affected by carcinoma cells. The mass screening for brain and breast tumor cells through non-invasive imaging is a prerequisite for detection at an early stage and further treatment. Tumor detection in the brain and breast is realized by a new morphological imaging modality known as diffuse optical tomography. Diffuse optical tomography is a non-invasive, non-ionizing, functional imaging model which is worth employing in Near-Infra-Red (NIR) wavelengths 700–1100 nm. The NIR light from the laser source illuminated the soft tissues of the brain from different locations. The light propagated through the tissue was measured using multiple photodetectors located on the surface of the phantom. Biological tissue strongly scatters at NIR wavelengths in diffuse optical tomography, which makes the tissue parameters at the boundary suffer from a highly nonlinear problem^1^. Experimental system^2^ is supported by passing an ultra-short pulse in the time domain or continuous intensity modulation in the frequency domain. Reconstructed image^3^ in spatial distribution with tissue parameters can be related directly to substantial properties^4^, such as blood and tissue oxygenation state. Image reconstruction from optical properties of tissue^5^ in diffuse optical tomography solves two individual problems, namely the forward and inverse problems. Forward problem^6^ predicts the light distribution at the detectors using light dissemination through the tissue. Inverse problem^7^ estimates the optical tissue properties, which reduces the similarity^8^ between the experimental and model-predicted measurements. The robust scattering characteristic of tissues in near-infrared wavelength ranges (700–1100) nm impairs the reconstructed picture quality, resulting in poor resolution. Because light scattering is strong, photons follow random trajectories. Further, it suggests that reconstruction is challenging since the arbitrary optical coefficient distribution results in an ill-posed issue. Thus, regularisation allows computing modeling 9 to solve the inverse issue but not the forward model. As a result, we use the regression model in Response Surface Methodology (RSM), particularly the ANOVA (Analysis of Variance) approach, to address the forward model problem by improving the experimental design. The associated study explains Culver's Diffuse Optical Tomography optimization approaches. Corlu et al. Yalavarthy et al., Xu et al., Chen, and Dehghani Chen et al., as well as Karkala et al.

The foremost aim of RSM is to use a sequence of designed experiments to obtain an optimal response. RSM is practical^9^, economical, and relatively easy for modeling, examination, and improving the experimental setup. The arithmetic prototype developed by RSM, then its competence is cross-verified by the Analysis of Variance (ANOVA) technique^10,11^. Response surface method^12^ is a collection of mathematical and statistical analysis^13^ of problems, wherein numerous unconditional variables x_1_,x_2_,…x_k_ called factors are impacted by a conditional variable ‘y’ called a response, and its objective is to optimize the result. The response surface methodology can be expressed as

1 $$y=f({x}_{1};{x}_{2};.....{x}_{k})$$

The unconditional variables are assumed to be continuous and controlled with a minor error to optimize the response variable y^14^. The resultant or the conditional variable is accepted as a random variable. The choice of experimental design radically influences the efficiency of the response surface analysis^15^. Central Composite Design (CCD)^16^ and Box–Behnken Design (BBD) are the categories of RSM^17^ chosen for our experimental design. Central Composite Design is realistic for a detailed forecast of all response variable averages concerning quantities measured during experiments. Box–Behnken Design^18^ is generally used to perform non-sequential experiments. These methodologies developed a second-order quadratic relationship between the experimental factors and responses. First and second-order coefficients are estimated efficiently from the design. Compared to the Central Composite design, the Box–Behnken design demands a lesser numeral of design points with a low cost to run.

Axial points are not present in Box–Behnken design^19^; thus, it is positive that all design points^20^ fall within the safe operating zone. The fitted second-order polynomial regression model, recognized as the quadratic model in RSM evaluation is used to approximate the response y^21^. The quadratic model is specified as

2 $$y={a}_{0}+\sum \limits_{i=1}^{4}{a}_{i}{x}_{i}+\sum \limits_{i=1}^{4}{a}_{ii}{x}_{i}^{2}+\sum \limits_{i\langle j}^{4}{a}_{ij}{x}_{i}{x}_{j}+\epsilon$$

where y is the answer (conditional variable), x_i_ is the input factors (unconditional variable), a_0_ is the constant regression coefficient, a_i_ is the first order coefficient, a_ii_ is the pure quadratic coefficient, and a_ij_ is the interaction term coefficient. $\epsilon$ is a random error that is assumed to be individually allocated. The Eq. (2) is valid until x_i_ is equal to x_k_.

3 $$\widehat{y}=y-\epsilon$$

The estimated response $\widehat{y}$ is constructed on the second-order model. Accurate predictions occur from the second-order response model owing to the influence of a single factor; quadratic term and their interaction effects are studied.

## Related works

Optimization in Diffuse Optical Tomography is demanding since it employs a variety of Diffuse Optical Tomography devices. Measurements are performed in a diversity of fields, including time domain, frequency domain, and continuous wave, using various measurement geometries, sampling densities, regularisation methods, and inversion techniques with varying signal-to-noise levels. Culver et al.^22^ states a numerical singular value analysis for a linear Diffuse Optical Tomography. The forward solution provides an obvious relation between signal, noise, regularization, and resolution in Diffuse Optical Tomography systems. Corlu et al.^23^ investigates wavelength-conditional tissue properties. They present a multispectral method for the reconstruction of tissue chromophore concentrations. The reconstructed variables are wavelength-conditional, and this approach effectively reduces the number of unknowns and produces better constrained on the inverse problem. The optimal wavelengths are 780 nm and 850 nm, which were investigated and proved in their experimental setup. Therefore, we chose these wavelengths for the experimental design of the forward model. Typically, there are three kinds of lasers, specifically red, blue, and green, where green and blue are consumed in industrial applications. Red lasers are used for biomedical applications as they are harmless. Xu et al.^24^ optimizes the fiber positions on the skull, and the hypothesis was examined on the head. They hypothesized the position of optical fibers on the side of the skull nearest to the brain is superior to arranging them likewise spaced around the entire head to maximize the sensitivity of brain tissue. Yalavarthy et al.^25^ specifically worked on mesh resolution in both the forward and inverse calculations. They investigated that quantitative accuracy increases with a better number of quantities in circular tomography imaging. Chen and Chen^26^ have used Cramer-Rao lower bound analysis to optimize source-detector arrangements. The quantitative estimation of lower bounds was constructed by certain parameters, such as reconstructed perturbation depths at different noise levels, which are estimated directly without solving the inverse problem. Dehghani et al.^27^ presented numerical simulations using the Finite Element Method on an adult head. Diffuse optical tomography was evaluated with different source-detector distances ranging from 1.3 to 5.5 cm; such hypothetical systems had higher sensitivity and imaging depths. Karkala et al.^28^ provided an optimum data-collection strategy that designs the data resolution matrix. They provided specific information about individual quantities, and their choice of using unconditional measurements does not compromise the image quality. A novel method of optimization of experimental design analysis for the Diffuse Optical Tomography system was accomplished using Regression analysis, introduced by the author outage the other optimization techniques by reducing error percentage.

## Experimental analysis and design

**Figure 1:**
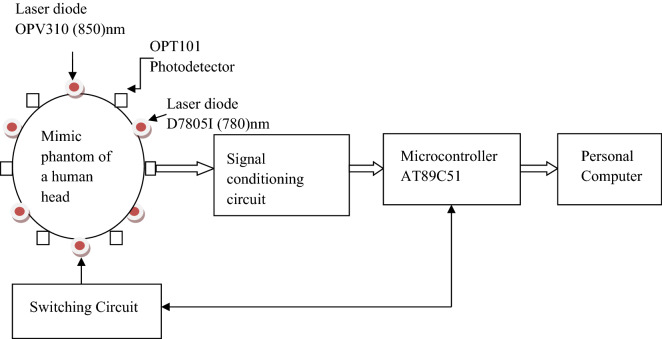
Diffuse optical tomography experimental model.

**Figure 2:**
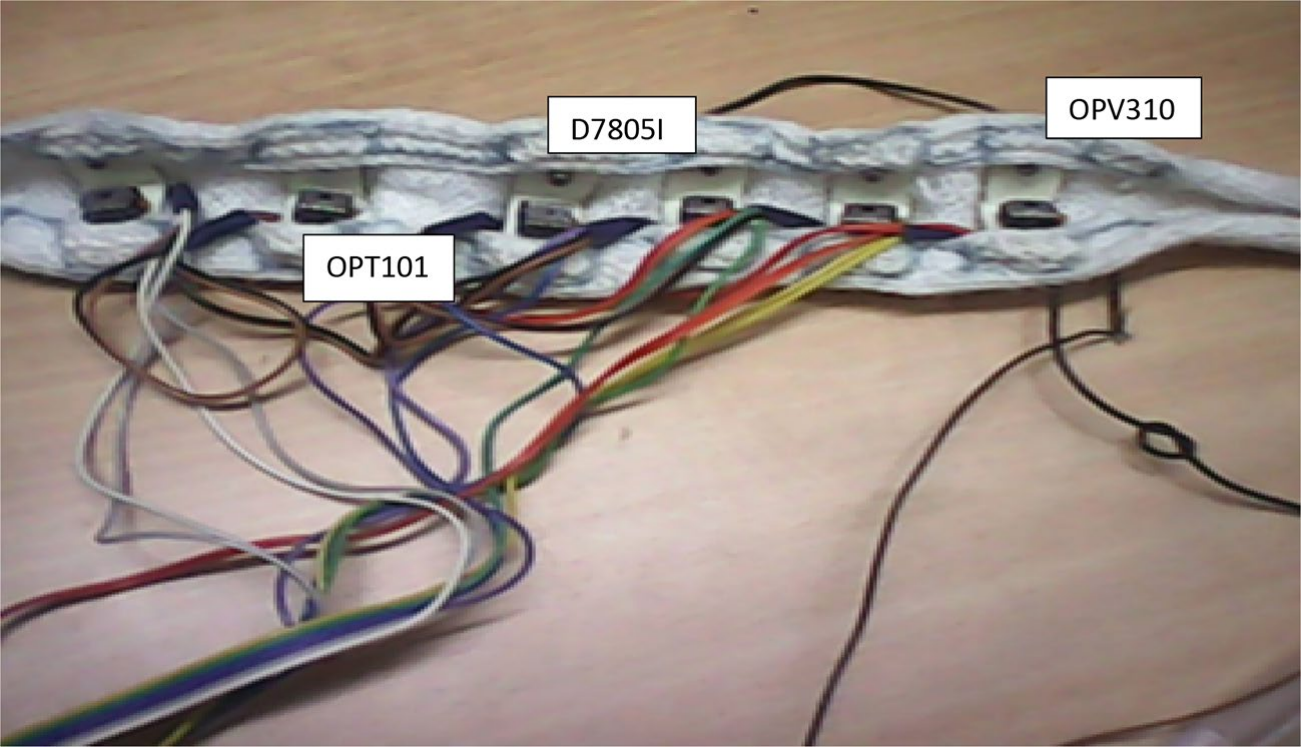
Laser sources and photo-detectors.

**Figure 3:**
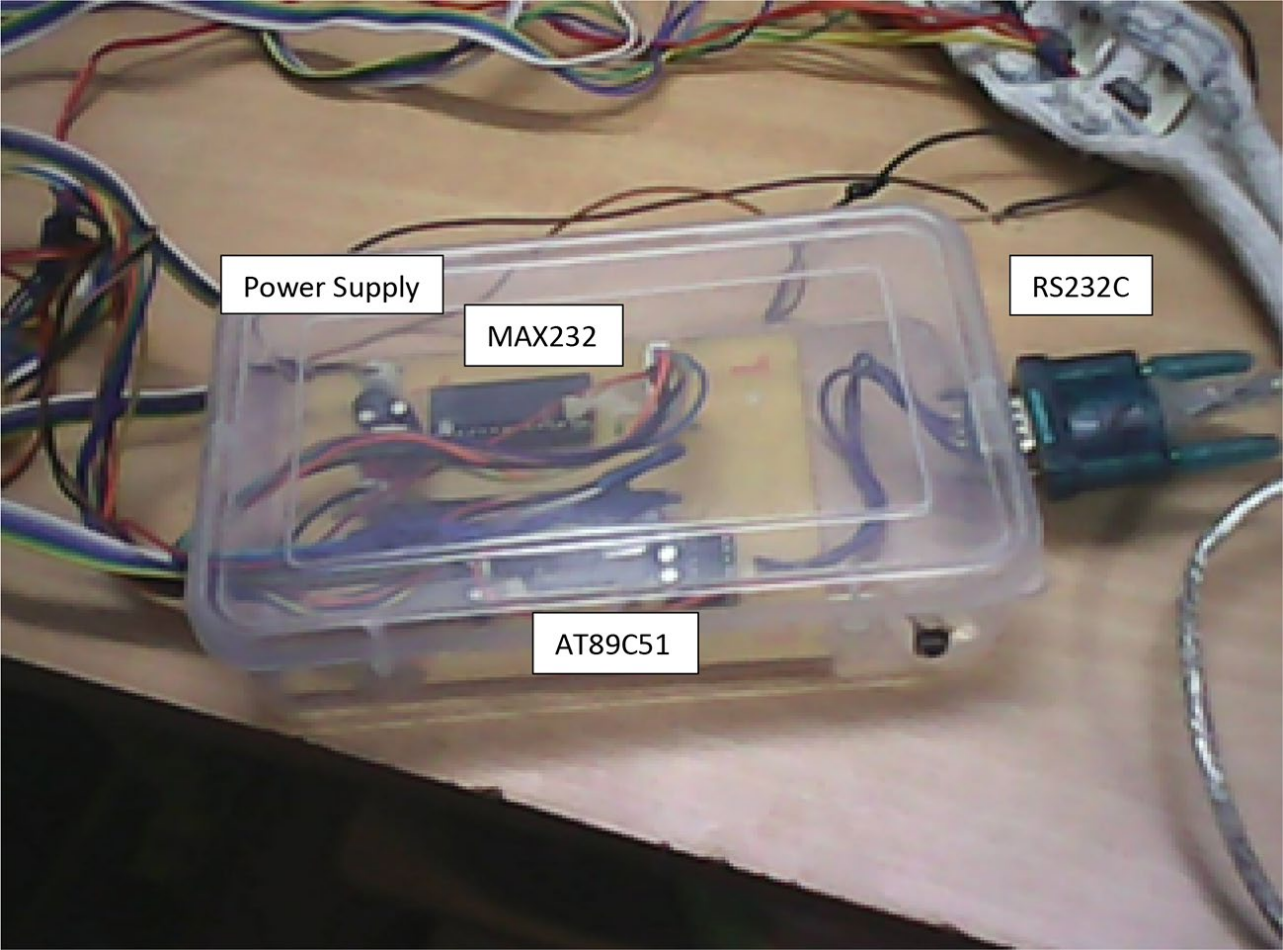
Switching and signal processing circuit.

The Diffuse Optical Tomography is a time-resolved experimental setup, as shown in Fig. 1. block diagram comprises six sets of laser diodes besides photo-detectors mounted on the mimic human head through a headband, and its implementation is exhibited in Fig. 2^29^. The phantom tested in human brain soft tissue is a mimic phantom. The OPV310 (850 nm) and D7805I (780 nm) 28 laser diodes are utilized, by a switching period of 3.3 ms. The laser diode operates within the 1.1 MHz and 1.2 MHz RF ranges, respectively. At NIR wavelengths, the innocuous red laser sources cause an incident in brain tissue. To exclude the crosstalk, OPT101 photo-detectors were arranged beside the six sources with an optimal separation of 2 cm. The OPT101 photodiode's trans-impedance amplifier boosts output voltage linearly as a function of illumination strength. Figure 3 depicts the shift of the laser-diode collection operated by the AT89C51 microcontroller, which in turn triggers the shift period by an operating indicator through MAX232. MAX232 is exhausted, to transmit data from the photodetector to the microcontroller; and from the microcontroller to the laser diode power source. Human cell tissue is not exercised in the study; instead, deformable Tissue Mimicking Materials (TMM), namely Polymethyl methacrylate (PMMA) and resins, are involved instead of human cells. TMMs match the absorption and scattering properties of soft tissues staunchly, hence, we employed them in the study. The geometry of the mimic phantom taken for study follows elliptical which is analogous to human brain features.

The occurrence voltage of OPV310 was 2.2 V, and D7805I was 3.5 V besides a limit of 1.1 mW and 5 mW of power. To attain, elevated rectilinear results at low dark currents, the photodiode operates in photo-conductive mode. The low pass filter in the signal processing circuit filters the noise voltages from the photo-diode. The filtered photodiode voltage is forwarded via RS232C to the ongoing interface of a personal computer, and^30^ becomes accumulated on the MATLAB workstation. The Lambert–Beer law similarity was deployed on the recorded photo-diode voltages. MATLAB 2013a was used to determine the absorption coefficient (mua cm^−1^), reduced scattering coefficient (mus cm^−1^), and photon flux (phi in arbitrary unit (a.u.)). The phantom picture was recreated through mua, mus, and phi computed by MATLAB, and suffers^31^ on dimensional outcome owing to forward challenge in the investigational model. Therefore, the Response Surface Methodology was related to the system design and analyzed using Box–Behnken Design.

A three-level Box–Behnken Design (BBD)^32^ of trials were tested with three unconditional variables which were coded to experimental values. The design was composed of 18 factorial designs (runs 1–18), 6 midpoints, and a group of points two-faced at the midpoint of every boundary of the multifaceted block that outlines the target area. The investigational data was analyzed^33^ using statistical methods, namely regression which appropriates the experimental design to obtain the optimum photonic flux. The nonlinear computer generated a quadratic model^34^ for three-level designs is given as

4 $$\begin{aligned} y & ={a}_{0}+{a}_{1}{x}_{1}+{a}_{2}{x}_{2}+{a}_{3}{x}_{3}+{a}_{12}{x}_{1}{x}_{2}+{a}_{13}{x}_{1}{x}_{3} \\ & \quad +{a}_{23}{x}_{2}{x}_{3}+{a}_{11}{x}_{1}^{2}+{a}_{22}{x}_{2}^{2}+{a}_{33}{x}_{3}^{2}+\epsilon \end{aligned}$$

$y$ is measured response^35^, ${a}_{0}$ is the intercept, ${a}_{1}$ to ${a}_{33}$ regression factors, ${x}_{1},{x}_{2},{x}_{3}$ are the implicit level of individual variables where the input voltages of 850 nm laser diode, 780 nm laser diode and spacing between the array. The unconditional and conditional variables used in the design were listed in Table 1.

**Table 1:** Box–Behnken experimental design variables.

Unconditional variables	Levels used (coded)			
	Units	Low (− 1)	Medium (0)	High (+ 1)
850 nm x1	volts	1.9	2.05	2.2
780 nm x2	volts	1.6	2	2.4
Space x3	cm	2	3	4
Conditional variables				
Photodetector Ph1	volts			
Photodetector Ph1	volts			
Photodetector Ph2	volts			
Photodetector Ph3	volts			
Photodetector Ph4	volts			
Photodetector Ph5	volts			
Photodetector Ph6	volts			
Absorption coefficient	cm^−1^			
Scattering coefficient	cm^−1^			
Photonic flux	a.u.			

The design response of the conditional variables for each input (unconditional) variable is demonstrated in Table 1. The unconditional input variable low, medium, and high-level ranges with units are specified. The experimental analysis is presented in Table 2. which displays the response of photodetector voltages (Ph1–Ph6), obtained from the experimental setup. The results based on response voltages will be optimized, by adjusting optical parameters such as the absorption coefficient (mua), reduced scattering coefficient (mus), and photonic flux (phi). The design matrix was constructed, on the experimental result, for each run of the recorded factors. Minimizing the result of inconsistency in the detected result was owing to irrelevant elements; the experiments were arbitrarily conducted.

**Table 2:** Design matrix concerning each response variable.

Experiment no	Factors			Response								
	Laser source		Space, cm x3	Photo-detector, volts						Absorption and reduced Scattering coefficient cm^−1^		Optical flux, phi a.u.
	850 nm x_1_	780 nm x_2_		Ph_1_	Ph_2_	Ph_3_	Ph_4_	Ph_5_	Ph_6_	mua	mus	
1	1.90	1.60	3	27.93	25.185	25.304	28.41	25.258	28.236	0.0908357	3.72849	0
2	2.20	1.60	3	26.592	28.905	28.852	25.987	28.95	28.563	0.0907061	3.70844	5.21822E−016
3	1.9	2.40	3	26.827	29.888	28.236	25.963	29.235	28.963	0.0905769	3.68851	1.16486E−015
4	2.20	2.40	3	26.818	26	26.987	25.872	25.21	25.389	0.0904481	3.66872	1.80328E−015
5	1.9	2.0	3	29.485	25.409	28.236	27.478	27.63	29.89	0.0903196	3.64906	2.43333E−015
6	2.20	2.0	2	27.382	26.483	26.963	26.963	26.358	29.963	0.0901915	3.62953	3.05431E−015
7	1.9	2.0	2	28.547	28.811	27.952	27.235	27.698	25.365	0.0900637	3.61013	3.66606E−015
8	2.20	2.0	4	27.328	27.34	26.563	29.689	25.954	27.365	0.0899364	3.59086	4.2686E−015
9	2.05	1.6	4	25.483	28.489	25.365	29.87	26.36	29.298	0.0898093	3.57171	4.862E−015
10	2.05	2.4	2	26.201	27.321	26.956	28.41	27.365	28.862	0.0896827	3.55269	5.44633E−015
11	2.05	1.6	2	29.017	25.594	29.985	25.456	29.63	29.986	0.0895563	3.53379	6.02171E−015
12	2.05	2.4	4	25.943	29.583	26.456	25.658	28.654	28.365	0.0894304	3.51502	6.58826E−015
13	2.05	2.0	4	27.333	27.291	29.562	28.562	25.963	26.258	0.0893048	3.49637	7.14607E−015
14	2.05	2.0	3	25.865	29.9	25.963	25.265	28.21	29.412	0.0891795	3.47784	7.69527E−015
15	2.05	2.0	3	25.491	28.594	25.562	29.789	26.63	25.589	0.0890546	3.45943	8.23597E−015
16	2.05	2.0	3	28.944	25.094	27.561	27.563	29.96	28.987	0.0889301	3.44114	8.76828E−015
17	2.05	2.0	3	26.4	28.287	28.506	27.985	28.639	28.41	0.0888059	3.42297	9.29232E−015
18	2.05	2.0	3	29.295	25.345	29.963	29.258	25.368	25.365	0.088682	3.40492	9.80819E−015

### Analysis of ANOVA

The test significance of the regression model, individual model coefficient, and test for lack of fit is summarized in ANOVA Tables 3, 4, and 5. The result summary shows the quadratic example response was probabilistically substantial under two dissimilar conditions. The model terms are noteworthy at a 95% significance level. To investigate the dependability and accuracy of the standard, the integrity of fitting was computed from R^2^ (coefficient of correlation) and CV (Coefficient of variation). Degrees of freedom were expressed as the numeral values that change unconditionally from each other. The regressive exclusion process spontaneously reduces the relations that are not substantial; thus subsequent ANOVA^36^ table is associated with a reduced Quadratic model for absorption coefficient (mua), reduced scattering coefficient (mus), and photonic flux (phi).

**Table 3:** ANOVA for response Surface Quadratic Model of mua.

Source	Sum of squares	Df	Mean square	F value	p-value prob > F
Model	7.496E−006	6	1.249E−006	49.04	< 0.0001
A-A	3.301E−008	1	3.301E−008	1.3	0.2791
B-B	7.401E−008	1	7.401E−008	2.91	0.1163
C-C	1.291E−007	1	1.291E−007	5.07	0.0911
A^2^	5.076E−006	1	5.076E−006	199.25	< 0.0001
B^2^	1.420E−006	1	1.420E−006	55.73	< 0.0001
C^2^	1.392E−008	1	1.392E−008	0.55	0.4753
Residual	2.802E−007	8	2.547E−008		
Lack of fit	8.732E−009	3	1.455E−009	0.027	0.9998
Pure error	2.715E−007	5	5.430E−008		
Cor total	7.776E−006	17

**Table 4:** ANOVA for Response Surface Quadratic Model of mus.

Source	Sum of squares	Df	Mean square	F value	p-value Prob > F
Model	0.17	6	0.028	51.4	< 0.0001
A-A	7.733E−004	1	7.733E−004	1.04	0.2616
B-B	1.725E−003	1	1.725E−003	3.13	0.1048
C-C	2.934E−003	1	2.934E−003	5.31	0.0417
A^2^	0.12	1	0.12	208.30	< 0.0001
B^2^	0.032	1	0.032	58.11	< 0.0001
C^2^	2.202E−004	1	2.202E−004	0.40	0.5406
Residual	6.073E−003	11	5.521E−004		
Lack of fit	2.201E−004	6	3.669E−005	0.031	0.997
Pure error	5.853E−003	5	1.171E−003		
Cor total	0.18	17

**Table 5:** ANOVA for Response Surface Quadratic Model of phi.

Source	Sum of squares	Df	Mean square	F value	p-value prob > F
Model	1.239E−014	6	2.056E−015	43.25	< 0.0001
A-A	2.195E−016	1	2.195E−016	4.62	0.0547
B-B	4.711E−016	1	4.711E−016	9.91	0.0093
C-C	1.668E−016	1	1.668E−016	3.51	0.0878
A^2^	7.902E−015	1	7.902E−015	166.26	< 0.0001
B^2^	2.641E−015	1	2.641E−015	55.56	< 0.0001
C^2^	2.906E−016	1	2.906E−016	6.11	0.0310
Residual	5.228E−016	11	4.753E−017		
Lack of fit	3.757E−016	6	6.261E−016	2.13	0.2124
Pure error	1.471E−016	5	2.942E−017		
Cor total	1.286E−014	17			

In the model table, the probability better than the F (probability > F) parameter is less than 0.05, indicating a substantial model since it specifies that the model terms have a substantial response. Model terms A is the modulation voltage of 850 nm, B is the modulation voltage of 780 nm, and C is the spacing between Laser Diodes. C, A^2^, and B^2^ are the substantial model terms. The principal result of second-order wavelength is that extremely trivial element exists along with photonic flux. Design-Expert software suggests that a quadratic model offers a satisfactory fit, and the design is obtained to have an insubstantial Lack of fit.

### Absorption coefficient (mua)

Table 3 Model F-value of 49.04 denotes that the design is substantial, and the Model value is around 0.01%. In line with a random value, this value is well-built, and standards with “probability > F” less than 0.05 imply that the model terms are substantial. The model terms, in this case, C, A^2^, and B^2^ are substantial. Values higher than 0.1000 specifies the design terms are insubstantial^37^. The F-value of 0.027 denotes the lack of fit is irrelevant compared to theoretical error. If the lack of fit value is considerable, it could not occur due to noise. Non-substantial lack of fit is good since we want the model to fit. “Pred-R-squared” of 0.9452 is in rational treaty with “Adj-R-Squared” of 0.9443. Adequate accuracy determines the signal-to-noise ratio and a factor higher than 4 is suitable, whereas a ratio of 18.178 denotes a satisfactory indication. The value of R^2^ is 96.40%, and the adjusted R^2^ is 94.43%, which means the regression model delivers an outstanding elucidation on the correlation among the unconventional variables (factors) and results.

Model P-value is smaller than 0.05 ((i.e.) $\alpha =0.05$ or 95% confidence), thus the design is regarded as probabilistically substantial. The quadratic model for the absorption coefficient regarding coded elements is set as

5 $$\begin{aligned} mua & =0.089-6.424E-005*A-9.618E-005*B \\ & \quad -1.270E-004*C+1.079E- 003 * {A}^{2} +5.704E \\ & \quad -004 * {B}^{2}+5.647E-005 * {C}^{2} \end{aligned}$$

### Reduced scattering coefficient (mus)

Table 4 Model F-value 51.4 denotes it is substantial, and the model terms are C, A^2^, and B2. The lack of fit F-value 0.031 denotes it is irrelevant to the theoretical inaccuracy. The "R-squared" suitable value is 0.9654; "Pred-R-squared" of 0.9470 is of a rational treaty with “Adj-R-squared” of 0.9465. The P-value is smaller than 0.0001; therefore, the model reliability is rationalized. The quadratic model for reduced scattering coefficient regarding coded elements is given as

6 $$\begin{aligned} mus & =3.45-9.831E-003*A-0.015*B-0.019*C \\ & \quad +0.16 * {A}^{2}+0.086 * {B}^{2}+7.104E-003 * {C}^{2}\end{aligned}$$

### Photonic flux (phi)

Table 5 Model F-value 43.25 denotes it is substantial, and the model terms are B, A^2^, B^2^, and C^2^. The lack of fit F-value 2.13 specifies that it is irrelevant to the theoretical inaccuracy. The "R-squared" suitable value is 0.9593; "Pred-R-squared" of 0.8666 is of a rational treaty with “Adj-R-squared” of 0.9372. The P-value is smaller than 0.0001; therefore, the model reliability is rationalized. The quadratic model for reduced scattering coefficient regarding coded elements is set as

7 $$\begin{aligned} ArcSin(Sqrt(phi)) & =9.201E-008+5.238E-009*A \\ & \quad +7.674E-009*B+4.566E-009*C-4.7255E-008*{A}^{2} \\ & \quad -2.460E-008*{B}^{2}+8.160E-009*{C}^{2}\end{aligned}$$

### Response surface analysis design

**Figure 4:**
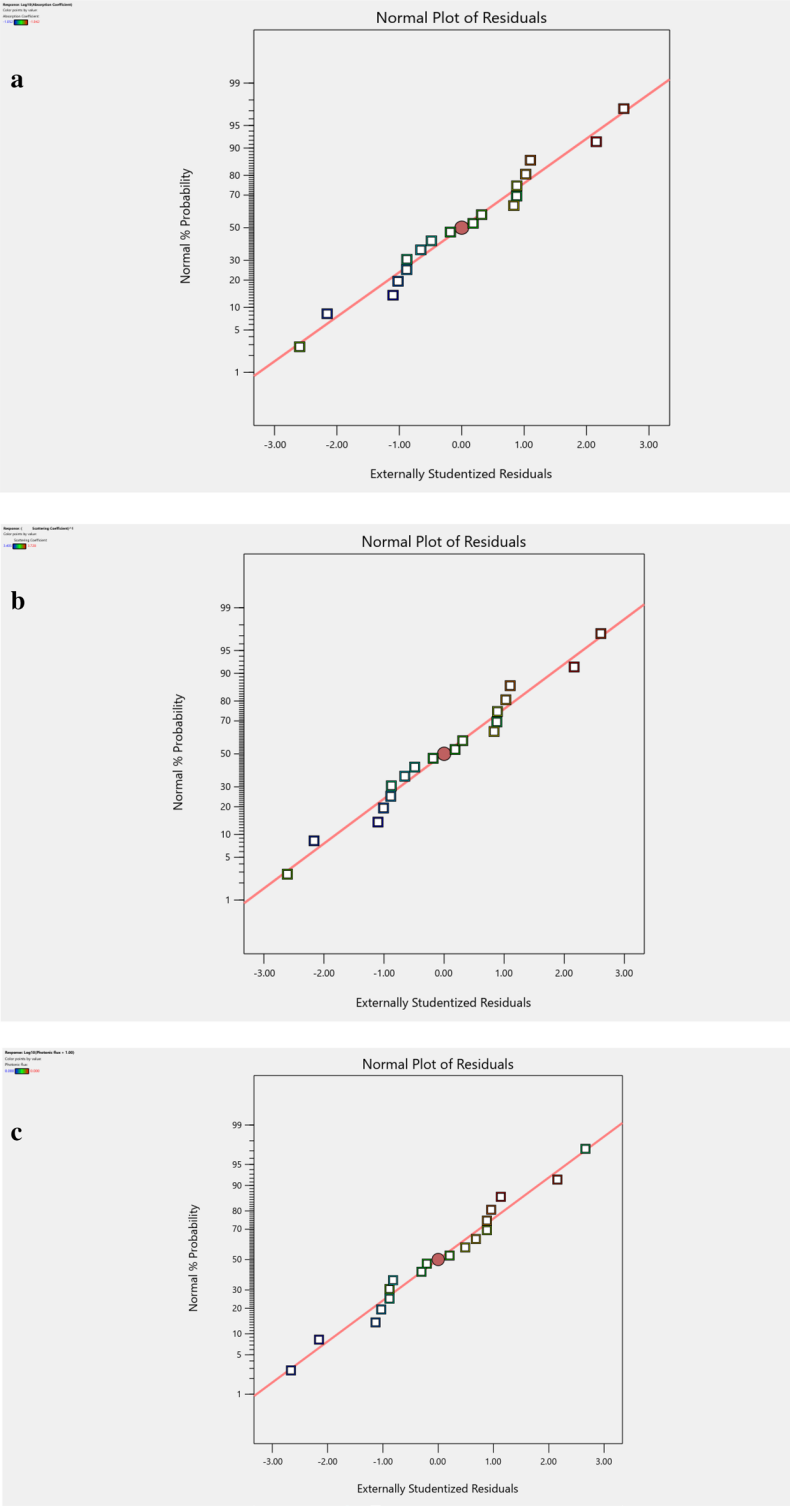
(**a**) The normal probability plot of residuals for mua—checks the mua data points are linear and follow the straight line (normal). (**b**) The Normal Probability plot of residuals for mus—checks the mus data points are linear and follow the straight line (normal). (**c**) The Normal Probability plot of residuals for phi—checks the phi data points are linear and follow the straight line (normal).

Figure 4a–c display the normal probability design of remainders declining at a rectilinear line, which denotes that the faults are typically and unconditionally allocated. To compute the efficacy of the investigational model, the first test error terms ${{e}_{i}}^{^{\prime}}s$ was assumed to be typically and individually scattered with zero mean and variance ${s}^{2}$. The regularity in the hypothesis was suited to the Residual chart near the 45° line. The error obtained is the dissimilarity relating the experimental value ${y}_{i}$ and the equivalent fixed value $\widehat{y}$. (i.e.) ${e}_{i}={y}_{i}-{\widehat{y}}_{i}$.

**Figure 5:**
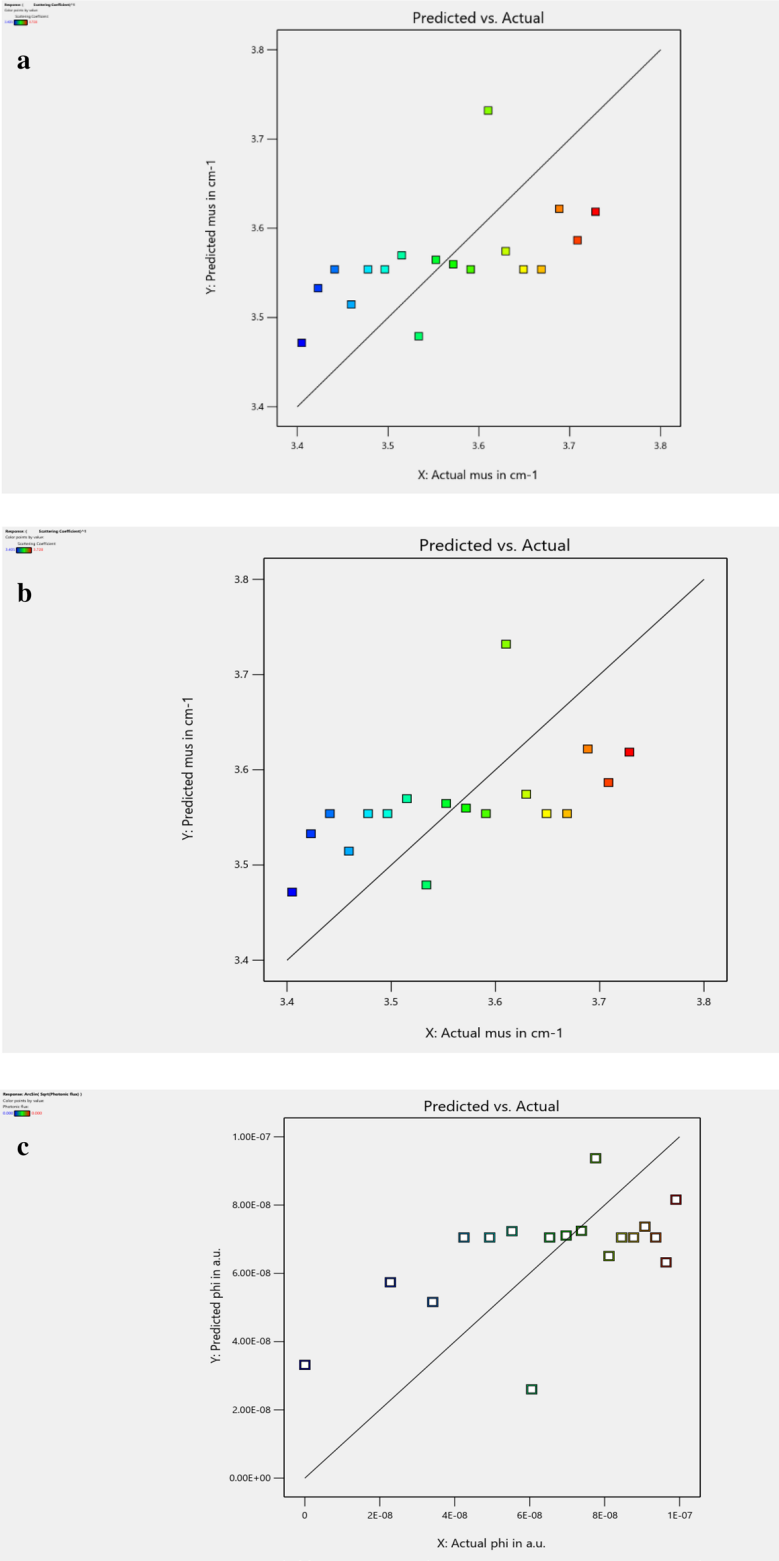
(**a**) Actual versus the Predicted value of mua—checks precise fit of actual response and prediction of mua for a set of values. (**b**) Actual versus the Predicted value of mus—checks precise fit of actual response and prediction of mus for a set of values. (**c**) Actual versus the predicted value of phi—checks precise fit of actual response and prediction of phi for a set of values.

Figure 5a–c depict the plot of the actual response value predicted for absorption coefficient (mua), reduced scattering coefficient (mus), and photonic flux (phi). The predicted values and the actual values were realized to be statistically similar. Each examined rate was assessed by the forecasted rate, which is specifically computed on the standard. All the points dropped uniformly on both sides of the 45° line. The regression standard satisfactorily suits the experimental results. The observations ${y}_{i}$ were typically and unconditionally distributed. The statistical regression model predicted provides optimum result; hence the experimental verification confirms the forecast value to be optimum.

### 2D contour and 3D surface plots

**Figure 6:**
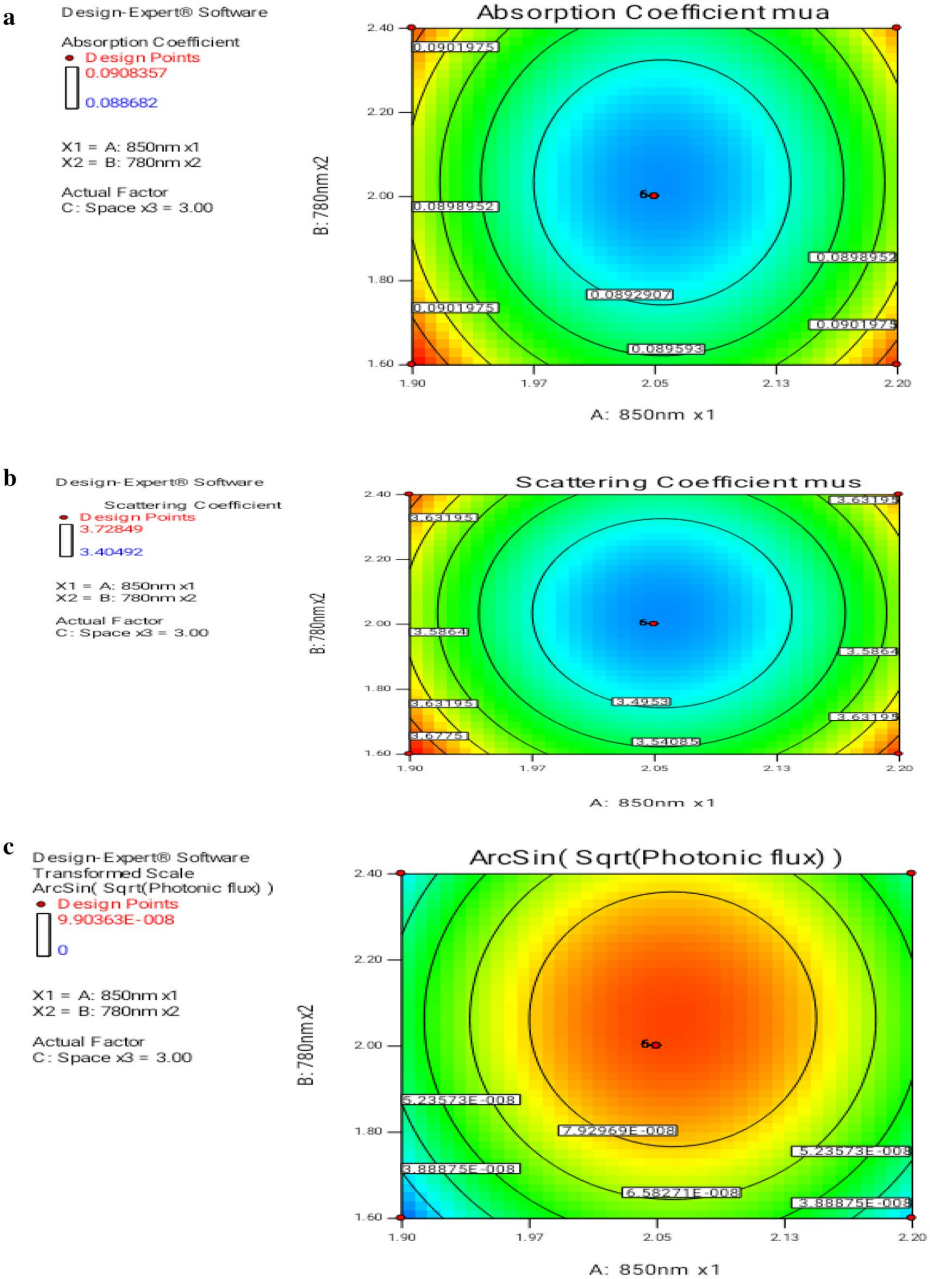
(**a**) Contour plot of two-factor interaction for mua—2D design produces mua response on variation in numeric input elements. (**b**) Contour plot of two-factor interaction for mus—2D design produces mus response on variation in numeric input factors. (**c**) Contour plot of two-factor interaction for phi—2D design produces phi response on variation in numeric input elements.

Contour plots shown in Fig. 6a–c were 2D illustrations of results designed for certain elements. 2D contour plots, which are bound to be elliptical, show substantial results in the optimization of the experimental setup. The primary goal based on the model was to find the adaptable target rates when the result is minimized. Every curve characterizes an unbounded quantity of probable permutations of the test variables, besides others kept at zero limits. In this contour diagram, the highest anticipated value was characterized by a tiny eclipse, and the elliptical contour measures the ideal relationships between unconditional variables.

The optimum value from the 2D contours was projected when the input voltages to laser diodes 850 nm and 780 nm were 2.05 and 2 V, respectively. The spacing between the arrays was 3 cm, the predicted values for absorption coefficient (mua) were 0.08899, the reduced scattering coefficient (mus) was 3.45044, and the optical flux phi was 9.6101E−08. The interaction between the input parameter voltages was realized to be less profound and linked to spacing which contributes to optimum prediction.

**Figure 7:**
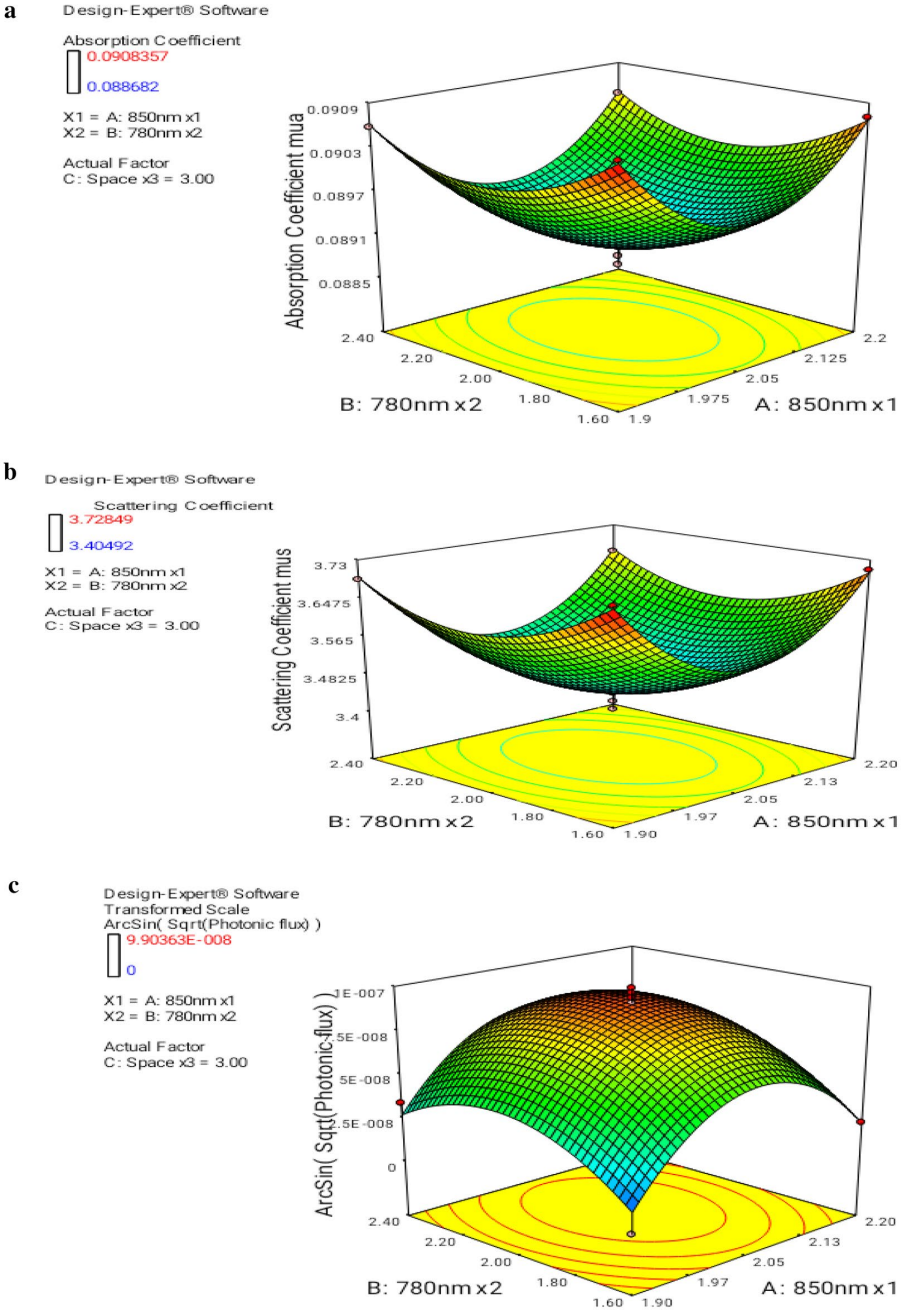
(**a**) 3D plot interaction of A and B for mua—displays the response mua based on the interaction of three numeric factors. (**b**) 3D plot interaction of A and B for mus—displays the results of mus based on the interaction of three numeric factors. (**c**) 3D plot interaction of A and B for phi—displays the response phi based on the interaction of three numeric factors.

Figure 7a–c predict the 3D contour plots with input interaction factors of laser diode input voltage in the ranges of 1.9–2.2 V and 1.6–2.4 V. Typically the spacing is optimized to 3 cm to prevent the crosstalk of the signals transmitted from the laser diode array, and then scattered rays are detected by the photodetector array. 3D surface design for the attained response was illustrated by the typical polynomial functions measuring the variation of the response surface. 3D design explains the connection relating to the results (conditional variable) and factors (unconditional variables). The design met the optimum condition with lower and upper bound ranges.

Figure 7a depicts the output absorption coefficient mua, in the range of 0.0885 to 0.0909, which is acquired by varying A and B factors within the span. Based on the desirability, the upper bound has 99.9% of the maximum scale value of the plot.

In Fig. 7b, the scattering coefficient mus was measured in the range of 3.4 to 3.73, which is attained by varying the interaction factors with a spacing of 3 cm. The upper bound predicts the desirability range of 99.9%.

Figure 7c depicts the variation in photonic flux by the interaction of unconditional variables A and B. 3D plot interaction scales the upper and lower bound values as 1.19E−08 to 9.27E−08, exhibits the trend of variation of responses within the selected range of A, B, and C parameters and the influence of each parameter over another parameter. The region of optimum conditions was achieved with an upper bound value lying in the desirability range. The optimum value of photonic flux was 7.346E−08 which is in the desirability range of 99.9%.

### Desirability function

**Figure 8:**
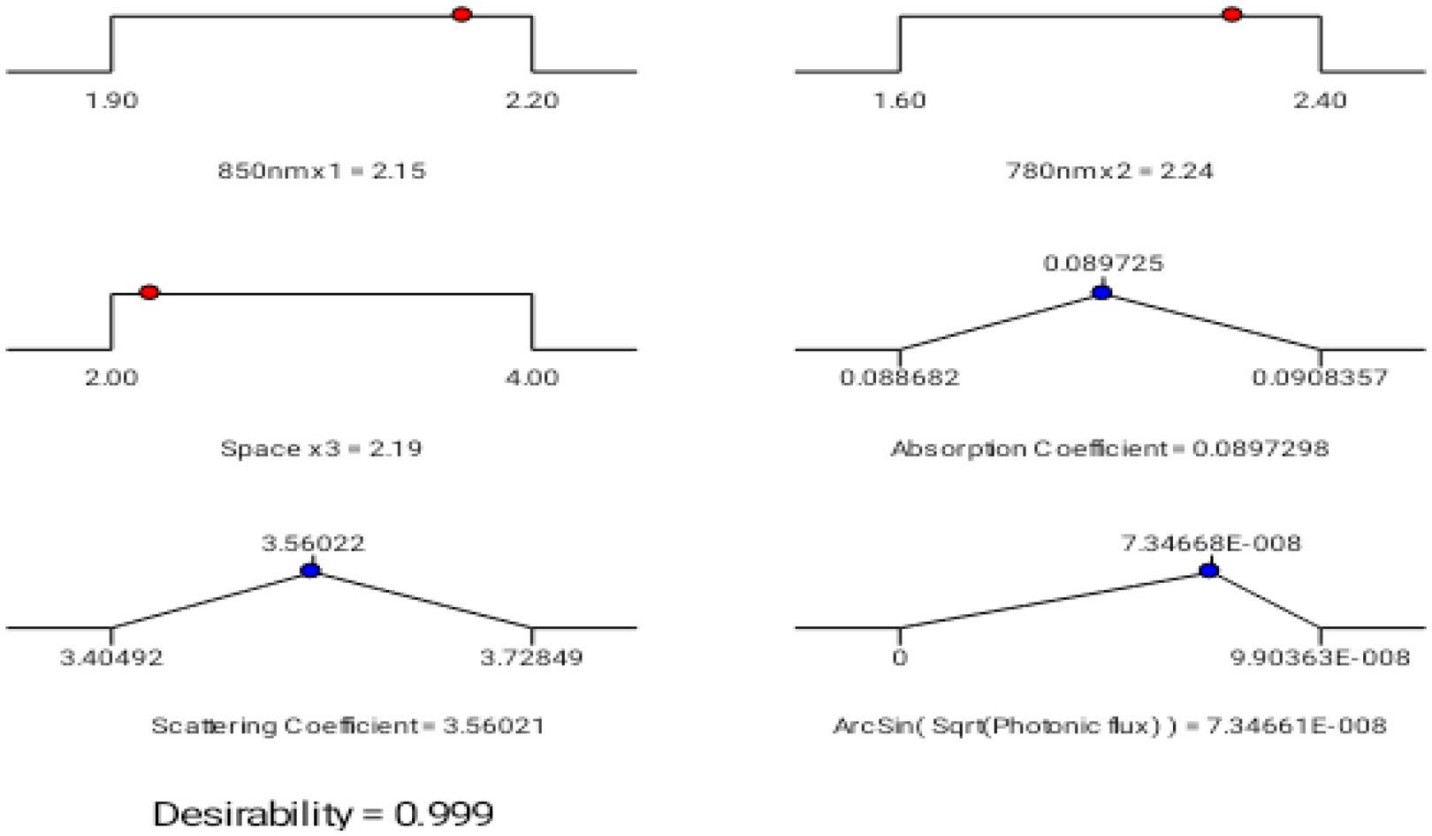
Ramp functional graph for optimum results—displays simple interpretation of input values to produce an optimized desirable response of 99.9%.

The desirability function $d({y}_{i})$ was worth optimizing the output parameter in the experimental result. The transfer function is given as

8 $${y}_{i}\left(x\right)={f}_{i}\left({x}_{1},{x}_{2},......{x}_{k}\right), \mathrm{i}=1,..,\mathrm{p}$$

where x_1_, x_2_,…x_k_ is total unconditional variables and i = 1,2,…p represent the multiple responses. The ${d}_{i}={d}_{i}\left({y}_{i}\right)={d}_{i}\left({y}_{i}\left(x\right)\right)$ desirability function will assign values between 0 and 1. Possible values of ${y}_{i}$ ${concerning \;d}_{i}\left({y}_{i}\right)=0 \; \& \; {d}_{i}({y}_{i})=1$, are the most desirable and undesirable values of ${y}_{i}$. Individual desirability $d({y}_{i})$ is associated with each requirement in ${y}_{i}$. The arithmetic mean of each discrete desirability function that exists in every multiple response problem is represented by desirability factor D. From, Fig. 8 Ramp functional chart provides the desirability result $d({y}_{i})=0.999$. Subsequently, the photonic flux of the forward model was organized to be maximized to obtain the high-resolution image in diffuse optical tomography^41,42^.

The desirability of 0.999 was obtained by setting input voltages of laser diodes 850 nm (x_1_) and 780 nm (x_2_) in ranges of 2.15 V and 2.24 V. Along with spacing (x_3_) of 2.19 cm, we obtain the optimum value of absorption coefficient mua in the target range as 0.0897298, scattering coefficient mus as 3.56021and photonic flux phi in the span of 7.34661E−008. In this experimental analysis, desirability must be close to 1 to maximize the outputs to reduce the forward model problem.

## Results and discussion

Confirmation experiments were accomplished to validate quadratic model adequacy. The residual error percentage was computed from the predicted value and actual experimented value with a prediction interval of 95%

9 $$Error\%=\frac{Experimental-PrPr e dicted}{PrPr e dicted} \times 100$$

The test results from experimental verification was displayed in Table 6; the error % lies between − 0.493% to 0.483% for absorption coefficient (mua), − 0.783% to 0.287% for reduced scattering coefficient (mus), and -0.751% to 0.651% for photonic flux (phi). The experimental test result lies within 95% of the confidence interval.

**Table 6:** Optimization Process for mua, mus, phi-RSM versus Experimental.

Factors			Response								
850 nm X_1_ volts	780 nm X_2_ volts	Space X_3_ cm	mua cm^−1^			mus cm^−1^			phi a.u.		
			Experimental	Predicted	Error%	Experimental	Predicted	Error %	Experimental	Predicted	Error %
1.94	1.83	2.88	0.089430	0.089	0.483	3.708437	3.70	0.228	9.27E−15	9.21E−15	0.651
**2.15**	**2.24**	**2.19**	**0.089729**	**0.090**	**− 0.301**	**3.560210**	**3.55**	**0.287**	**7.34E−08**	**7.33E−08**	**0.136**
2.05	2	3	0.090577	0.091	− 0.465	3.515017	3.51	0.142	9.25E−15	9.23E−15	0.216
2	2	3	0.090706	0.091	− 0.323	3.441142	3.45	− 0.256	8.97E−15	8.93E−15	0.447
1.93	2.16	3	0.089556	0.09	− 0.493	3.422974	3.45	− 0.783	9.3E−15	9.32E−15	− 0.214
2	1.6	3	0.089683	0.09	− 0.353	3.53379	3.54	− 0.175	9.24E−15	9.31E−15	− 0.751

Significant values are in bold.

**Figure 9:**
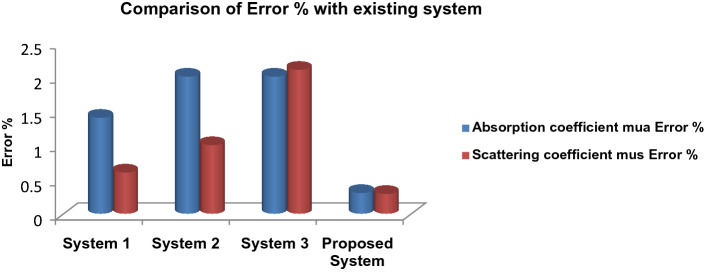
Comparison of error % with the existing system.

Table 7 compares the expected error percentage 36 figures to the three current 37 systems 38. The proposed system error % for Absorption coefficient mua was 0.301 and for scattering coefficient mus was 0.287. A performance comparison of the proposed system with the existing system is displayed in Fig. 9.

**Table 7:** Comparison of the proposed system with the existing system.

System	Optical parameters (cm^−1^)	Measured (cm^−1^)	Predicted (cm^−1^)	Error
Sultana et al.^38^	µ_s_^′^	11.57000	9.300	0.240
	µ_a_	0.036000	0.034	0.050
Ilias et al.^39^	µ_s_^′^	9.700000	9.300	0.040
	µ_a_	0.107000	0.034	2.100
Eiji and David^40^	µ_s_^′^	9.100000	9.300	0.210
	µ_a_	0.140000	0.034	3.110
**Optical property extraction system**	**µ**_**s**_^**′**^	**3.560210**	**3.550**	**0.287**
	**µ**_**a**_	**0.089729**	**0.090**	**0.301**

Significant values are in bold.

## Conclusion

The Box–Behnken architecture was exercised in an experimental program to maximize the forward model parameters. The response surface approach, which has been effective, may be used to access the three important Diffuse Optical Tomography input components. The factors were the input voltages of 850 nm (x_1_), 780 nm (x_2_), and space between the arrays (x_3_) in the forward model; the output response parameters were absorption coefficient (mua), reduced scattering coefficient (mus) and photonic flux (phi). The spacing (x_3_) between the array of laser diodes and the photo-detector has implications for determining the optimum values of the results. Statistical Regression model equations were obtained by the software package Design Expert 7.0. The experimental data was justified, by the competency of an analytical model. The forecast value and experimental value were realized to be in perfect agreement. The optimum process parameters were attained when the input voltages of a laser diode as 2.15 V, and 2.24 V, and the spacing was 2.19 cm. The optimum results were an absorption coefficient (mua) value of 0.089 cm^−1^, reduced scattering coefficient (mus) value of 3.5 cm^−1^, and photonic flux value7.3E−08a.u. The value of R^2^ (0.95) is close to 1, specifying an extraordinary measure of correspondence relating to the result and unconditional variables, which was exposed by five experimental verification responses displayed in Table 6. The optimized formulations produced experimental values for the response variables that are linearly close to the predictions. Figure 8 proves that our proposed system exhibits better performance in similarity with the existing system.

## Data Availability

The datasets used and analysed during the current study available from the corresponding author on reasonable request.
